# Stress Hyperglycemia Secondary to Abdominal Emergency Mimicking Diabetic Ketoacidosis in a Pediatric Patient

**DOI:** 10.7759/cureus.37505

**Published:** 2023-04-12

**Authors:** Ossma E Elbelihy, Hussein A Masalmeh, Muhammad Nauman Shah, Noman Khan, Amr A Gebril

**Affiliations:** 1 Emergency Medicine, Al Dhafra Hospital, Abu Dhabi, ARE; 2 Emergency Medicine, Madinat Zayed Hospital, Abu Dhabi, ARE; 3 Radiology, Aga Khan University Hospital, Karachi, PAK; 4 Emergency Medicine, NMC Royal Hospital, Abu Dhabi, ARE

**Keywords:** acidosis lactic, acute abdomen in children, surgical acute abdomen, non dka, dka

## Abstract

Abdominal pain in a pediatric patient with diabetic ketoacidosis (DKA) can be mistaken for surgical or septic causes of acute abdomen. Both DKA and surgical abdominal emergencies can cause lactic acidosis (LA), which makes it challenging to differentiate between them. Fluid therapy resulting in quick alleviation of metabolic acidosis could be a valuable sign in differentiating surgical abdomen from DKA. In this report, we present a case of the surgical abdomen with stress hyperglycemia that mimicked DKA.

## Introduction

Stress hyperglycemia refers to elevated levels of blood glucose in the absence of diabetes mellitus, resulting from stress, such as ischemia/infarction, sepsis, traumatic injuries, burns, or surgery [1]. Diabetic ketoacidosis (DKA) is an acute metabolic complication of diabetes mellitus, most commonly type 1 diabetes mellitus [2,3]. Tachypnea, abdominal pain, hyperglycemia, and anion gap metabolic acidosis in a pediatric patient is indicative of DKA [4]. However, other medical conditions such as abdominal surgical emergencies may mimic DKA clinically. We report a case of stress hyperglycemia in a patient with an acute abdominal emergency that mimicked DKA on the initial presentation. Evaluation of our patient was rendered more challenging due to the patient's disability, gaps in the provided history, late presentation, and difficult clinical examination. The patient was diagnosed with small bowel volvulus and underwent abdominal surgery, with subsequent uneventful recovery.

## Case presentation

An 11-year-old male patient presented to the emergency department accompanied by his father in the evening due to abdominal pain that had started in the morning. The patient's history provided by the father was inadequate, and he was unaware if the patient had any associated symptoms (e.g., vomiting, fever, or diarrhea). According to prior medical records, the child had multiple disabilities including atonic cerebral palsy, congenital deafness, and mutism, and was completely bedridden. The patient also had a history of patent ductus arteriosus, congenital hypertrophic pyloric stenosis, pulmonary hypertension, and bronchopulmonary dysplasia secondary to prolonged ICU stay for extreme prematurity. Additionally, there was a history of pyloromyotomy for hypertrophic pyloric stenosis in the second month of life and a history of bilateral inguinal hernia repair surgery at the age of six months. The patient’s pulmonary hypertension was managed medically with oxygen and continuous positive airway pressure.

On examination, the patient was toxic-appearing and in moderate pain. He was tachypneic and tachycardic. The patient was taking rapid shallow breaths; however, he had clear lungs on auscultation and had bilateral equal air entry. Cardiac auscultation revealed no abnormality. There was no murmur. The patient's abdomen appeared distended, with a tympanic sound on percussion. There was diffuse abdominal tenderness in four quadrants, but more pronounced in the epigastric region. His capillary refill was delayed. The patient was afebrile. Skin turgor was poor and mucous membranes were dry. His blood pressure was 91/70 mmHg with a pulse rate of 149 bpm and oxygen saturation of 95%.

Initial management of the patient included intravenous access with intravenous fluid and analgesia for pain relief. Samples were taken for laboratory investigations. Laboratory tests revealed an elevated white blood cell (WBC) count of 25 x 10^9^/L with a neutrophilic predominance of 89.2%. His C-reactive protein (CRP) was normal (2.1 mg/dL). Serum sodium concentration was 142 mEq/mol, potassium concentration was 3.2 mEq/mol, chloride concentration was 94 mEq/mol, and serum creatine was 110 micromol/L. Serum urea levels were 8.7 mmol/L. Venous blood gas (VBG) analysis revealed a low pH of 7.172, PaCO_2_ of 63.9, and an HCO_3_ level of 17.4 mg/dL. The lactate level was 8.9 mmol/L and the calculated anion gap was 30. Fingerstick blood glucose was 330 mg/dL. The overall metabolic picture was suggestive of a mixed disorder of high anion gap metabolic and respiratory acidosis.

**Figure 1:**
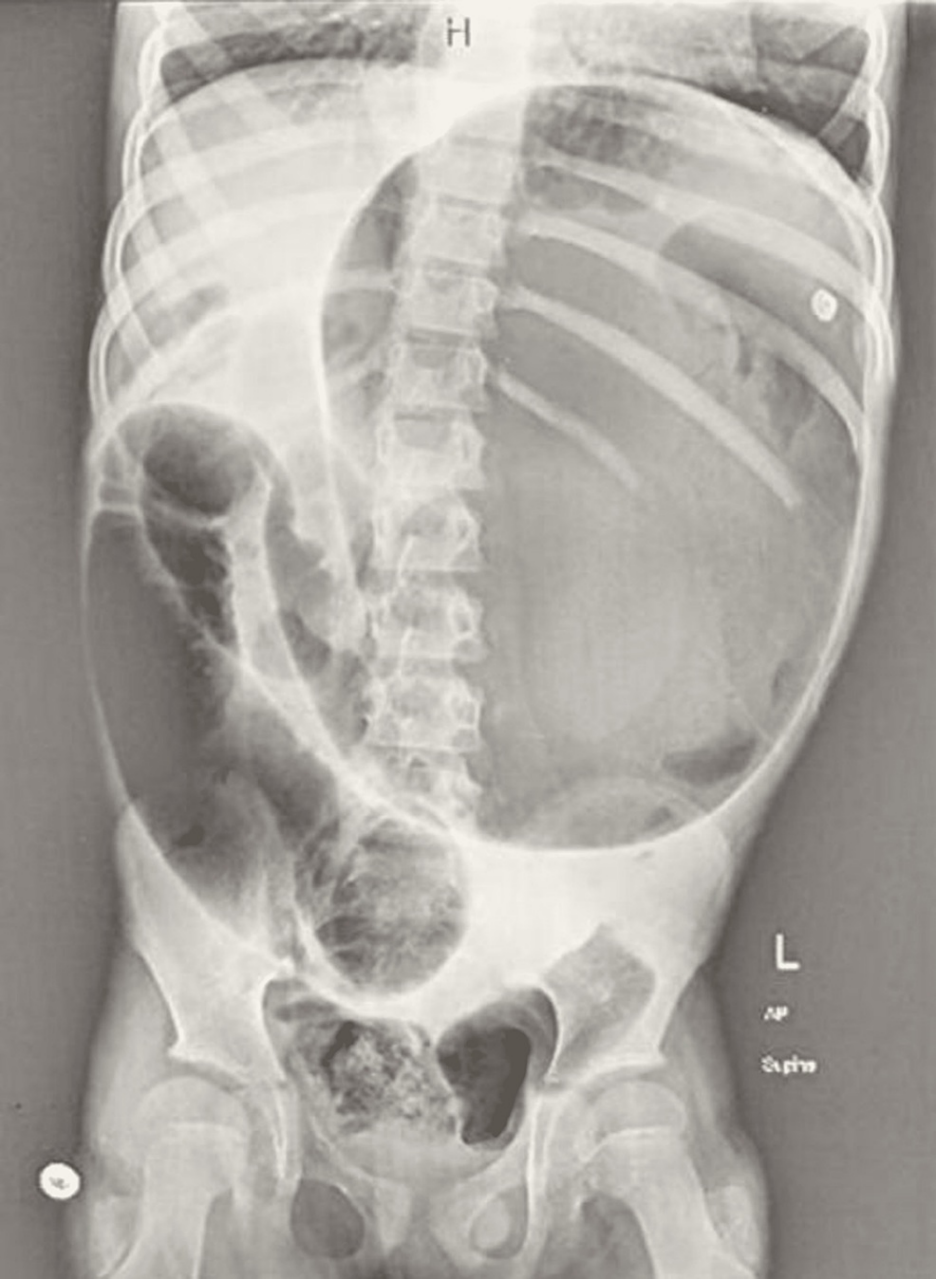
Plain radiograph of the abdomen Significant distension of the stomach and adjacent duodenum is seen. A small amount of air and fecal material can also be appreciated in the sigmoid colon

**Figure 2:**
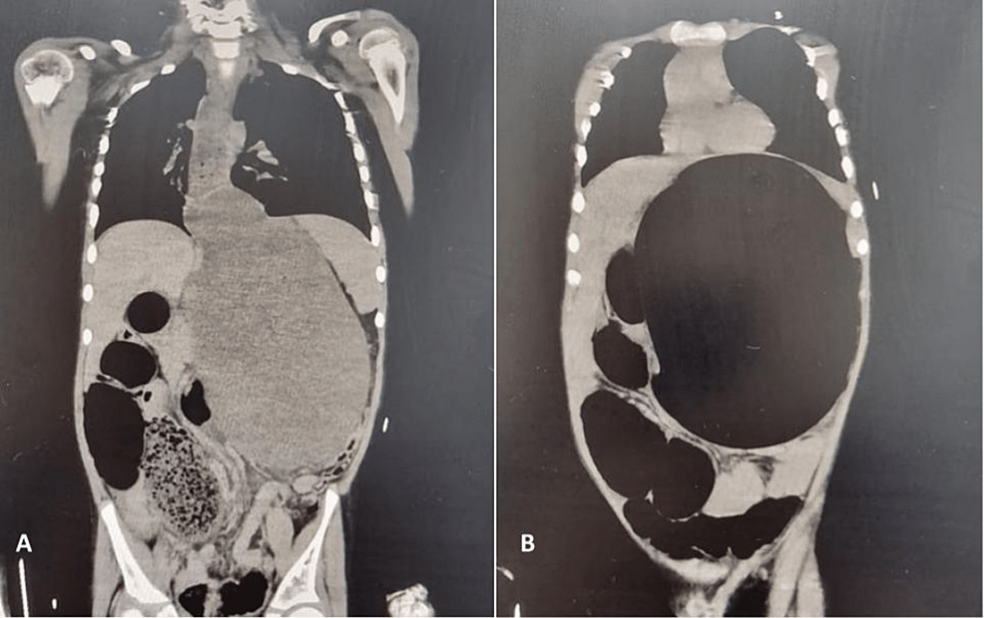
Noncontrast CT abdomen (coronal sections through the abdomen) Significant distention of the stomach and distal esophagus can be seen. Air is also seen in the small bowel CT: computed tomography

Due to severe abdominal pain, the possibility of acute abdomen was considered along with DKA. A plain radiograph of the abdomen revealed significant distension of the stomach and duodenum (Figure 1). Noncontrast CT abdomen revealed significant distention of the stomach and duodenum with an air-fluid level (Figure 2). The large bowel was unremarkable except for a moderate amount of air in the sigmoid colon. A nasogastric tube was inserted for decompression. Gradually, the patient showed marked clinical improvement with the normalization of capillary refill and resolution of tachypnea. Subsequent arterial blood gas (ABG) analysis revealed a pH of 7.37, PaCO2 of 30.2, and HCO3 of 20. Fingerstick blood glucose levels dropped to 180 mg/dl and lactate levels dropped to 3.2. There was a normalization of the anion gap at this point.

Given the rapid improvement following nasogastric decompression and fluid resuscitation, the clinical picture suggested stress hyperglycemia and lactic acidosis (LA) secondary to an acute abdominal emergency that mimicked DKA.

Broad-spectrum antibiotics were initiated with maintenance fluids. A Foley catheter was placed and the patient was transferred to another facility with pediatric surgery and pediatric ICU facilities. On clinical suspicion of adhesive intestinal obstruction and possible midgut volvulus, the patient underwent laparotomy on the second day of initial presentation. Surgical findings were consistent with the clinical suspicion and included volvulus secondary to a band. A bowel segment of ischemic with small bowel was noted 25 cm distal to the duodenojejunal (DJ) flexure. This band and ischemic bowel segments were resected, and end-to-end jejuno-jejunal (JJ) anastomosis was performed. The patient had an uneventful immediate postop stay in the PICU and was shifted to the ward after four days. Following an uneventful postop course, the patient was discharged after two weeks in stable condition. The patient was seen in the outpatient clinic three weeks postoperatively.

## Discussion

Abdominal pain is a common symptom of DKA, found in 40-75% of cases [4]. Its prevalence tends to increase as arterial pH and serum bicarbonate levels decrease. Additionally, around 12% of DKA patients with abdominal pain experience abdominal rebound tenderness, which suggests the presence of an acute abdomen. Although metabolic stabilization can often lead to clinical improvement, up to 35% of patients have an underlying cause of abdominal pain that precipitates the DKA, such as pancreatitis, hepatitis, pyelonephritis, pelvic inflammatory disease, or gastritis. In some cases (6%), surgical intervention is necessary, due to conditions such as Fournier’s necrotizing fasciitis, acute cholecystitis, appendicitis, and perineal abscess [4].

DKA can sometimes imitate infections, making it challenging to differentiate between septic and non-septic inflammatory responses [5]. The standard clinical indicators of systemic inflammatory response syndrome are not particularly specific, and suspicion of infection cannot be solely relied upon. Symptoms such as tachycardia and polypnea can be attributed to DKA, while hypothermia, fever, and abnormal white blood cell counts are typically used to evaluate septic status. However, none of these indicators have been shown to be effective in distinguishing between infected and non-infected patients with DKA.

LA is classified as a high anion gap metabolic acidosis, with an anion gap greater than 10. The cut-off level for blood lactate concentration varies between different studies, with some setting it at ≥2.5 mmol/l, while others have set it at >5.0 mmol/l (normal range: 0.4-1.2), in addition to meeting other criteria [6-10]. Numerous studies have demonstrated that lactate levels can be used as a predictor of illness severity in various critical conditions, such as sepsis, burns, ST-elevation myocardial infarction, post-cardiac arrest, and trauma [11-13].

Differentiating between DKA and LA that arises from a surgical abdominal emergency and is accompanied by stress hyperglycemia can be difficult, leading to delayed diagnosis and increased mortality. In patients with DKA, excessive fluid administration can be harmful, whereas those with septicemia or shock require aggressive fluid replacement therapy. However, failure to distinguish between the two conditions can prevent adequate fluid administration in patients with septicemia or shock, exacerbating the clinical situation.

During periods of severe stress, stress hyperglycemia and insulin resistance are natural responses that have been preserved throughout evolution to aid in the survival of the host [5]. Attempting to artificially normalize blood glucose levels can therefore impede immune and cerebral function during a crisis [14,15]. While high levels of lactic acid should be taken into account, they cannot be solely relied upon to differentiate between these scenarios. The prevalence of LA in the DKA population is a matter of controversy, with some reports suggesting that elevations in blood lactate concentration are common in patients with hypovolemia, hypotension, and hyperventilation, all of which are frequently present in patients with DKA. Other reports suggest that DKA and LA are distinct conditions that rarely occur together.

According to Kitabchi et al., measuring the concentration of lactate in the blood can easily confirm a diagnosis of LA (if it is greater than 5 mmol/L) since DKA patients rarely have such high levels of serum lactate [16]. This implies that DKA and LA should be distinguished from each other since LA is infrequently associated with DKA. A study by Watkins et al. found that individuals with diabetes who have LA typically have a severe underlying condition and a poor prognosis [17].

Although previous studies suggest that LA in DKA is rare and associated with poor outcomes, some studies have found that it is more common than previously thought and not linked to increased length of stay in the ICU or mortality. The fact that lactate levels are positively correlated with glucose suggests that altered glucose metabolism, in addition to hypoperfusion, may contribute to LA in DKA [6]. In a recent study, the presence of significant LA in DKA patients did not result in a longer hospital stay or complications typically associated with DKA, such as respiratory failure, cardiac arrhythmia, or cerebral edema. Additionally, no fatalities were reported during the study. According to recent research, increased lactate levels in DKA may not be a reliable predictor of the severity of illness, mortality, or other associated conditions. Instead, it may be an adaptive response that provides alternative fuel sources to vital organs when the body is in a hypoinsulinemic state [18].

While a high level of lactic acid should be definitely taken into consideration, it is not sufficient as a sole indicator to differentiate between the two scenarios. Serial abdominal examination and appropriate imaging techniques can be beneficial in identifying surgical causes of abdominal pain in both situations, which require early intervention. In our case, the rapid improvement of metabolic acidosis and the normalization of the anion gap with fluid therapy served as a potential tool for differentiation.

## Conclusions

Abdominal pain is a common symptom observed in DKA and can be mistaken for surgical or septic causes of acute abdomen. Both surgical abdominal emergency and DKA can cause LA, making it unreliable in differentiating DKA from septic or surgical causes of acute abdomen. Therefore, serial abdominal exams and proper imaging are critical in identifying surgical causes of the acute abdomen that require immediate intervention. However, rapid improvement of metabolic acidosis with fluid therapy may serve as a useful tool in distinguishing acute abdomen from DKA.
